# Medical care ideals among urban and rural residents in Thailand: a qualitative study

**DOI:** 10.1186/s12939-015-0292-6

**Published:** 2016-01-05

**Authors:** Tomoki Ikai, Saowalak Yamtree, Takuji Takemoto, Taro Tamura, Hitomi Kanayama, Kazuhiro Sato, Yukinori Kusaka, Hiroyuki Hayashi, Hidekazu Terasawa

**Affiliations:** Division of Primary Health Care, Faculty of Medical Sciences, University of Fukui, 23-3 Matsuoka-shimoaizuki, Eiheiji-cho, Yoshida-gun, Fukui 9101193 Japan; Faculty of Nursing, College of Asian Scholars, Khon Kaen, Thailand; Headquarters for Innovative Society-Academia Cooperation, University of Fukui, Fukui, Japan; Division of Environmental Health, Department of International and Social Medicine, Faculty of Medical Sciences, University of Fukui, Fukui, Japan; Division of General Internal Medicine, Faculty of Medical Sciences, University of Fukui, Fukui, Japan; Division of Promotion of Community Medicine, Faculty of Medical Sciences, University of Fukui, Fukui, Japan

**Keywords:** Thailand, Medical care ideals, Urban, Rural, Grounded theory

## Abstract

**Background:**

Health care is generally considered to be more highly valued in urban areas than in rural areas. However, studies have reported that there is no difference in the health care values of urban and rural areas in the Kingdom of Thailand, with some studies even indicating that these values are stronger in rural areas. We, therefore, conducted interviews and implemented a qualitative investigation and analysis aimed at elucidating ideals relating to the medical environment among the Kingdom’s urban and rural citizens.

**Methods:**

The study targeted Thai citizens residing in urban and rural areas. The city of Khon Kaen, located in Khon Kaen Province in northeastern Thailand, was selected as the urban area for the study. We selected Donyang village, located in the same province, as the rural study area. In July 2014, we conducted semi-structured group interviews, applying the Constructivist Grounded Theory (CGT) analytical approach.

**Results:**

We interviewed ten people in Khon Kaen (the urban area) and seven people from Donyang village (the rural area). Five major and distinctive themes emerged from the interviews. These were: *locally appropriate standards of medical care*, *support for local lifestyles*, *satisfaction with local medical personnel*, *healthy lifestyles that do not rely on medical services*, and *desire for regional autonomy/desire to serve the region in terms of medical care*. All of these themes were evident in both study areas. Thus, rather than relying on advanced medical services, both urban and rural Thai citizens expressed the desire to continue living within communities (considered as “families”), contributing to them, and tending to all of their health care needs within their communities.

**Conclusions:**

This study revealed five common themes relating to forms of medical care regarded as ideal among urban and rural citizens of Thailand. Its findings could potentially have important implications for areas characterized by urban–rural inequities relating to the accessibility and utilization of medical services.

## Background

### Study context and objectives

Urban and rural/remote communities differ in their utilization of medical care. Specifically, health care values are generally considered to be more pronounced in urban areas than in rural areas [1–13]. For example, Sewo *et al*. [14] have reported a higher total mean score for the quality of life (QOL) among urban residents compared with those in rural areas. However, Yiengprugsawan *et al*. [15] found that Thai Mental Health Indicators (TMHIs) of cohorts in urban and rural areas in Thailand did not differ significantly from each other. Thus, previous studies have found that health care values do not differ between Thai urban and rural areas, with some indicating that a higher value is placed on these values in rural areas [16–18].

Consequently, we conducted interviews and a qualitative investigation and analysis aimed at elucidating ideals relating to the medical environment among Thai urban and rural citizens. The insights and findings gained in the context of Thailand will, we hope, contribute to resolving worldwide inequity in health care.

### Study setting

The Kingdom of Thailand is situated in continental Southeast Asia (just north of the equator) and is part of the Indo–China Peninsula. Thailand covers an area of about 514,000 km^2^. Between its furthest points, from north to south, the distance is 1,860 km. The country comprises 76 provinces, with mountainous terrain and basins in the north and a fertile delta area around the Chao Phraya River in the central region. The southern region is surrounded by the South China Sea and the Indian Ocean. The Thai ethnic group is dominant in the country, with most citizens being Buddhist [19]. Approximately 40 % of Thai citizens are engaged in agriculture, which accounts for just 12 % of the GDP. Moreover, approximately 15 % of citizens are engaged in manufacturing, which accounts for 34 % of the GDP and nearly 90 % of exports [20]. Thailand’s population comprises 64 million people. The ratio of doctors to the total population is approximately 0.32 for every 1000 people—approximately one-tenth of the average of OECD countries, that is, 3.3 per 1000 people. Further, more than 100,000 professional nurses contribute to primary health care. Approximately one million health care volunteers, who have undergone training, constitute additional medical personnel, mainly in farming villages. Thailand’s citizens as well as medical personnel are highly concentrated in the capital, Bangkok, whereas medical care personnel are scarce in other areas. Thus, in 2009, while there were 1.77 doctors for every 1,000 people in Bangkok, the corresponding figure for the country’s northeastern region was just 0.35 doctors for every 1,000 people. Similarly, the population/bed ratio in Bangkok was 312 and that of the northeast region was 779. The ratio of medical devices such as CT and MRI machines evidenced the same regional difference. Although the average lifespan in the country was 71.7 years from 2005 to 2010, the morbidity rates for urban and rural regions differed. Provinces with ample health resources (low population/doctor and population/bed ratios) had higher medical facility utilization rates. Additionally, these indices evidenced a clear bias in relation to rusticity in each region [19].

For this study, we selected the city of Khon Kaen, located in Khon Kaen Province in northeastern Thailand as the urban area, and Donyang village, located in the same province, as its rural counterpart. Khon Kaen has a population of approximately 120,000 people, making it one of the largest cities in northeastern Thailand. The city is located at the intersection of two important Southeast Asian transportation routes, namely, an east–west route linking Myanmar and Vietnam and a north–south route linking Bangkok and Laos. Donyang village has a population of 2,600 people, mostly engaged in agriculture. It is located approximately 15 km from the outskirts of Khon Kaen, and can be accessed by car from this city in approximately 30 minutes. There is no hospital in Donyang village; only a primary care clinic staffed by a doctor, who is only available on Mondays, and community nurses who are available on other days.

In 2001, Thailand launched a national health insurance program to provide universal health insurance coverage, popularly known as “30-baht health care” (health insurance with 30 baht deductible for every consultation or for hospitalization). More than 90 % of Thai citizens are enrolled in health insurance [21]. While socioeconomic disparities exist in both urban and rural areas [22], under the universal health insurance coverage scheme, primary health care access among socioeconomically disadvantaged individuals has improved dramatically [23].

## Methods

### Participants

The current study targeted residents of urban and rural areas of Thailand, namely, the city of Khon Kaen and Donyang village. We contacted nurses engaged in health management in the village, explaining the general purpose of the study to them in advance and informing them of the interview date. On the day of the interview, we read out a document to the gathered participants that explained the study’s purpose and significance, its methods, duration, how participants were selected, logistical considerations, and publication venue. This document was then shared with them. We confirmed their agreement to participate in the study, using a written informed consent form, before conducting the interviews.

### Data collection

In July 2014, we conducted semi-structured interviews with the participants. All interviews were conducted with the facilitation of a local interpreter. To ensure consistency of interview content, smooth progression of the interviews, and collection of nonverbal sensory information, interviews were only conducted by the primary researcher using the same interpreter. We applied the interview guide, shown in Table 1. This was successfully used in a similar study conducted in Japan to clarify medical care ideals [24].

**Table 1:** Interview Guide

1	Out of all the medical care that you, your family, or acquaintances have received to date, what sort of medical care would you describe as “good” medical care? Why do you think it was good?
2	Conversely, what sort of medical care would you describe as “not good”? Why?
3	Do you think that the medical care you get in your own community is better than that in other communities? Do you think it is worse? What sort of communities do you think have worse medical care, and which do you think have better? Why do you think this?
4	What sort of medical care would you consider to be ideal for you?

Group interviews involving two to three people were conducted. These were considered as separate interviews if the group members changed. In accordance with the memos, and any other relevant information obtained such as “medical experiences affected attitude to medical care ideals,” we added interviewees who had undergone medical admission for our theoretical sampling. The interviews were held at public locations such as meeting halls and temples, or at participants’ homes. They were recorded with a digital recorder, and transcripts were produced based on the interpreter’s statements. Moreover, during the interviews, the appearance, attitude, and general impression obtained of each participant were recorded, and these were used for analyzing nonverbal data. We analyzed the interviews, by date, in chronological order. The participants could leave after we had confirmed that theoretical saturation had been reached in each area and that no new ideas could be obtained.

### Data analysis

The grounded theory research approach emphasizes inductive analysis. Deduction, which is the usual form of analytic thinking applied in medical research, proceeds from the general to the particular. It begins with pre-existing hypotheses or theories, and entails data collection aimed at testing these theories. Conversely, induction proceeds from the particular to the general, and entails the development of new theories or hypotheses based on many observations. The emphasis on induction within studies that apply grounded theory means that they tend to adopt a very open approach regarding the process being studied. A study based on grounded theory may evolve as the researchers become aware of what the study participants consider to be important [25]. For this study, we applied a Constructivist Grounded Theory (CGT) approach [26]. Grounded theory is a qualitative method of analysis proposed and developed by Glaser and Strauss [27–29]. Grounded theory does not adequately attend to the process of data production because of its prior objectivist empirical tradition. By contrast, data and analysis relating to the modified CGT approach formulated by Charmaz [30] focus on phenomena associated with research subjects, as constructed through experiences and relationships forged with researchers. The CGT approach assumes that data and theories are neither emergent nor discovered. Rather, they are mutually “constructed” by the researcher and the research participant. It emphasizes the importance of studying the extent to which the research subjects’ experiences are embedded in concepts that are less likely to surface such as positional networks, situations, and relationships. CGT has been recommended as an appropriate method for situations wherein researchers wish to understand the true feelings of participants in a variety of situations. It is best suited to addressing research questions that explore the complexities of cross-cultural issues and the abstract nature of the global mindset concept, along with the meanings assigned by participants to these issues as they frame them within the contexts of their own lives [31].

Based on the experiences, feelings, and attitudes of individual research participants, we elicited the elements of ideal medical care. We subsequently arranged these within storylines; repeated initial and focused coding, as well as sampling for each of the two areas; and elaborated on themes based on impressions, information, and memos obtained from the interviews. Analyses were performed for the new themes while repeatedly revisiting the original data and themes. Based on a focused consideration of data from one region, or alternatively, a consideration of data from another region, characteristic themes for the entire region were established for the theoretical coding. As the overall flow of the interviews and non-verbal information were required to accomplish this, a consistent approach was ensured through the performance of the analysis by just the primary researcher. The MAXQDA v.10 software (www.maxqda.com) was used to perform the analysis. MAXQDA is a convenient software package for conducting qualitative analysis of transcript data, enabling the aggregation of code frequency, data analysis by code segments and the creation of code matrices.

### Ethical approval

Ethical approval for this study was granted by the Ethics Committees, Faculty of Medical Sciences, University of Fukui (Ethics Hearing —26-56).

## Results

### Sample

The study included 17 participants (15 females), whose ages ranged from 41 to 83 years. Basic data for participants in each area, such as the number of participants, the proportion of males and females, education and income levels, and prior histories of medical care are shown in Table 2.

**Table 2:** Profiles of Participants Interviewed about Ideal Health Care in Thailand

Characteristics		Urban (*N* = 10)	Rural (*N* = 7)
Females		8	7
Mean age at interview		54.6	60.0
Highest level of education	Graduation from lower elementary division (Grade 4)	1 (10.0 %)	7 (57.1 %)
	Graduation from upper elementary division (Grade 7)	2 (20.0 %)	1 (14.3 %)
	Graduation from lower middle division (Grade 10)	2 (20.0 %)	0 (0.0 %)
	Graduation from upper middle division (Grade 12)	2 (20.0 %)	0 (0.0 %)
	Graduation from higher education (two years)	1 (10.0 %)	0 (0.0 %)
	Graduation from higher education (four years)	2 (20.0 %)	2 (28.6 %)
Household income	Less than 200,000 baht	3 (30.0 %)	1 (14.3 %)
	200,000 baht–400,000 baht	4 (40.0 %)	2 (28.6 %)
	400,000 baht–600,000 baht	0 (0.0 %)	2 (28.6 %)
	600,000 baht–800,000 baht	2 (20.0 %)	2 (28.6 %)
	800,000 baht or more	0 (0.0 %)	0 (0.0 %)
Number of respondents who visited a hospital or clinic on a regular basis (%)		8 (80.0 %)	5 (71.4 %)
Experience of hospital admissions (%)	Never	2 (20.0 %)	3 (42.8 %)
	1–2	4 (40.0 %)	1 (14.3 %)
	3–5	2 (20.0 %)	1 (14.3 %)
	6 times or more	2 (20.0 %)	2 (28.6 %)

### Themes

Five major themes were identified. These were: *locally appropriate standards of medical care*, *support for local lifestyles*, *satisfaction with local medical personnel*, *healthy lifestyles that do not rely on medical services*, and *desire for regional autonomy/desire to serve the region in terms of medical care*. All of these themes were observed in both study areas.

### Locally appropriate standards of medical care

Two focused codes were extracted for the theme, *locally appropriate standards of medical care*. These were: “the degree of advancement, degree of specialization, personnel, number of facilities, and medical devices met local needs” and “consultations were not refused,” Examples of relevant statements made by four participants in relation to this theme are listed below. Participant Rural02 described ideals relating to medical technology within institutions. Participant Rural05 opined that a minimum level of medical care was required in the area, entailing the provision of guaranteed consultations for citizens. Participant Urban01 believed that the personnel and facilities were not appropriate for the area. Last, according to Participant Urban06, a minimum level of service should be provided, and lack of human resources should be acknowledged. Further, citizens should be allowed to choose the medical institution for their treatment.

*We have two general hospitals nearby: Hospital A, which is public, and Hospital B, which is private. I think both of them have good systems. I think hospitals should have medical devices for examining and diagnosing illnesses. These hospitals have those. (Participant Rural02)*

*You won’t be refused a consultation at the public hospital. When you need something, you should at least be able to get a minimum level of service. (Participant Rural05)*

*I’ve heard that there are some places [hospitals] that don’t have enough space and personnel in the city, too. I’ve heard that sometimes the hospitals put patients in the hallways. They should have more employees and facilities, because there is a shortage of these in the area. (Participant Urban01)*

*There definitely are not enough doctors, and I don’t think there are enough [of] other medical personnel either. I’d like to be able to choose [the medical institution]. (Participant Urban06)*

### Support for local lifestyles

The theme of *support for local lifestyles* comprised ideals held by residents regarding the medical services that they required to be able to continue living in their respective areas. This theme included the following focused codes: “accessibility in terms of distance” and “fees for treatment that enables a sustainable lifestyle.” Three examples of statements made by participants are presented below. Participant Urban08 spoke about road conditions and the accessibility of hospitals, and Participant Rural03 spoke about the accessibility of nearby clinics. Participant Rural04 spoke about medical fees paid by patients themselves under the Thai medical system, as well as the ease of living.

*You can get there [to the hospital] in a few minutes by car, so it’s convenient. The roads are not bad [they are paved]. (Participant Urban08)*

*A primary health care clinic opened 5 km from here, three years ago, so it’s easier [to get a consultation]. Before, we had to go out of our way and travel very far. (Participant Rural03)*

*In Thailand, we have the 30-baht system, so most people can have a consultation for 30 baht. You can get aid if your income is low, so life is easy. (Participant Rural04)*

### Satisfaction with local medical personnel

This theme comprised two main vectors: distance and communication. The first vector, distance, indicated satisfaction relating to the presence of medical personnel such as community nurses and volunteers, as well as medical facilities in the vicinity. It was associated with focused codes such as “accessibility to medical personnel” and “comprehensiveness of medical care in the area.” The second vector, communication, indicated satisfaction based on the recognition that citizens were being watched over by medical personnel, or that they were understood by these personnel. Focused codes associated with this vector included “awareness of being watched over,” “kindness of medical personnel,” and “ease of exchanging information with medical personnel.” Four illustrative statements by participants are presented below. Participant Urban10 expressed satisfaction with the accessibility of medical care as volunteers made rounds to see patients. Participant Rural06 described an ideal situation of inpatient and outpatient treatment by medical personnel in the area. Participant Urban02 expressed satisfaction relating to the awareness of being watched over by citizen volunteers. Last, Participant Rural07 was satisfied with community nurses’ understanding of the needs of the area.

*Volunteers make rounds to see us and care about our health. Thanks to them, we can get detailed information about health in the area, so we trust them and rely on them. (Participant Urban10)*

*I’d be more satisfied if nearby primary care clinics would care for us even when we need inpatient treatment. I want to be cared for in the same area by the same nurses. I don’t want to go to the hospital. (Participant Rural06)*

*The volunteers care for us and understand us, so I’m satisfied. (Participant Urban02)*

*The community nurses really know everything about the area. They know who lives where, what illnesses they have, and what type of life they live. I think they might even know more than those of us who live here. (Participant Rural07)*

### Healthy lifestyles that do not rely on medical services

The theme of *healthy lifestyles that do not rely on medical services* focused not only on attempts to extend life and cure illnesses through medical services, but also on living in such a way that medical services were not required. It entailed focused codes such as “prevention and access to medical information for improving health” and “reliance on folk, traditional, and spiritual treatment methods.” Three relevant examples of statements by participants are presented here. Participant Urban05 described a cultural preference for traditional care over modern medicine, because citizens were not inclined to visit hospitals. Participant Rural01 was satisfied with medical treatment, because the participant was physically able to take care of herself. Participant Rural06 desired knowledge and guidance from medical personnel on health maintenance.

*Even when we don’t feel well, we don’t go to the hospital right away. That’s because of a foundation of cultural beliefs that folk treatments, or spiritual treatments such as religious purification, can cure illnesses. This may be why few people are inclined to go to the hospital. (Participant Urban05)*

*There is a clinic nearby, and we take care of our health on our own … I think the current medical environment is good enough. (Participant Rural01)*

*If we had doctors who would look more at the living environment, including prevention and improvement of health, our medical care would be better. (Participant Rural06)*

### Desire for regional autonomy/desire to serve the region in terms of medical care

The theme relating to *desire for regional autonomy/desire to serve the region in terms of medical care* is a particularly interesting one that was evident in focused codes such as “regional activities by community members (voluntary, care for the elderly),” “family unity in the area,” “desire to contribute to the area,” “being considerate of one another,” and “respect for ancestors.” Three related statements by participants are presented below. Participant Urban07 described a personal connection to the area, as well as the importance of contributing to the area. Participant Urban08 described care for the elderly and regional initiatives undertaken by local groups with government support. Participant Rural01 felt that activities by citizens in the area strengthened regional unity, contributing to the actualization of ideal medical care.

*Traditional Thai society involves an awareness of all the citizens in the area [being] relatives [one family]. The culture puts a high value on contributing to the family and the area, and we are taught not to forget this from childhood. We think the same way about implementing medical care. (Participant Urban07)*

*Everyone in the area gathers on Buddhist holidays to take care of the elderly. The elderly are the most highly valued people in the villages. The people apply for their own health promotion projects and take action with financial support from the government. (Participant Urban08)*

*I think that the activities by volunteers themselves strengthen regional unity. In cities, people are not around their families and there is a weak connection to the area. So probably people won’t cooperate with each other unless something brings them together. (Participant Rural01)*

## Discussion

Previous studies have generally indicated that there are disparities in accessibility and utilization of medical care between urban and rural areas [7–13]. Some studies have reported that there are variations in the medical environment between urban and rural areas in Thailand [23, 32–34]. We, therefore, investigated why, in contrast to these findings, our study did not find any difference between urban and rural areas in terms of medical care ideals held by their residents. One unique aspect of the identity lies in the lack of ideals relating to medical skills and procedures. Rather than desiring advanced medical technology, Thai citizens appear to prefer a healthy lifestyle that is not dependent upon medical services. Citizens emphasized their lifestyles in their areas over the nature of medical care in relation to themes such as *support for local lifestyles*, *satisfaction with local medical personnel*, and *desire for regional autonomy/desire to serve the region in terms of medical care.* In fact, they may not have understood the benefits of advanced medical care, as indicated by the following statement by Participant Rural05: “I don’t know what kind of services they provide at hospitals.” This view appears to be endorsed by Miedema, Easley, and Robinson based on their interviews of cancer patients in urban and rural areas [35]. These demonstrated the high expectations of urban residents relating to medical care and, conversely, the low expectations of residents of remote areas relating to such care, influencing their degree of satisfaction [35]. However, the reason for this finding was revealed through another theme examined in this study, namely, *desire for regional autonomy/desire to serve the region in terms of medical care*. As stated by Participant Urban07: “All the citizens in the area are related [one family].” There is a strong sense within the community of being a family, and in the past, this family was responsible for health care functions. Thus, the scope of existing criteria such as QOL for evaluating medical care appears to be limited. Martin *et al.* observed that QOL in Khon Kaen was strongly influenced by family, money, accommodation, and employment, in addition to health [36]. Yiengprugsawan *et al.* noted that in Thailand, the three most influential factors relating to happiness were the standard of living, future security, and life achievement [19]. In addition, Jongudomkarn and Camfield found that there were limitations relating to the quantitative measurement of the quality of health care using QOL, because Thai citizens emphasize basic criteria such as significant primary relationships [37]. This could explain the possible occurrence of shared ideals when citizens feel a sense of belonging, despite differences between urban and rural areas. Because basic criteria emphasized by Thai citizens, as identified in this study, can be explained by defining health literacy in terms of a family/society network, and health information [38], we feel that these are appropriate for the result of the current study.

Additionally, the findings of several previous studies match those of this study. Unlike in other countries, there is no difference in the physical health and happiness of urban and rural residents in Thailand [18]. Large-scale cohort studies have found no differences in the happiness of urban and rural residents in Thailand [15]. This explains the lack of differences in criteria for evaluating medical services, even though medical environments differ in Thailand. We should not, therefore, conclude that evaluations of medical services and views on medical care change based on accessibility and use of medical resources in Thailand.

This study had a number of limitations. First, the participants were not randomly sampled. Consequently, there may have been imbalances in relation to sex, age, academic history, income, and medical history among them. They cannot, therefore, be generalized. Sobieszczyk *et al.* have affirmed that no major differences exist regarding gender and well being among elderly people in Thailand [39]. By contrast, Sumngern *et al.* noted that there was a significant difference in the happiness of elderly people in urban and rural areas within the Thai province of Chonburi [40]. Additionally, Coronini-Cronberg *et al.* observed that even in an extremely impoverished area like Khon Kaen, health care was well-used [21], while Mordacci and Sobel found that personal views on health and medical history often influenced ideas and cultures on health [41]. However, the findings of these studies are not consistent, and we believe that our study appropriately reveals one aspect of healthcare in Thailand.

While this study targeted the areas of Khon Kaen and a nearby rural village, there is no evidence to support generalization of its findings as being representative of urban and rural areas across Thailand. Findings for provinces other than Khon Kaen may differ. Regional unity, accessibility, and use of health care facilities, would likely differ in a metropolitan area such as Bangkok, which has more migrants than Khon Kaen city, and in communities that are more rural in character than Donyang village, thus yielding different results. Future research will, therefore, need to gather data from a more diverse range of regions.

## Conclusion

This study has uncovered five prevalent themes relating to forms of medical care regarded as ideal by citizens in urban and rural areas of Thailand. These are: *locally appropriate standards of medical care*, *support for local lifestyles*, *satisfaction with local medical personnel*, *healthy lifestyles that do not rely on medical services*, and *desire for regional autonomy/desire to serve the region in terms of medical care*. Rather than relying on advanced medical services, citizens prefer to continue living in communities (which they regard as “families”), to contribute to their communities, and to meet all of their health care needs from within their communities. The findings of this study could potentially make a positive contribution in regions where there are inequities in the accessibility and use of medical services between urban and rural areas. A number of studies have observed that forms of social capital such as regional ties and trusting relationships have socio-epidemiological implications for health [42, 43]. We believe that our results will extend and deepen this body of knowledge.
